# Hydroxyl Conducting Hydrogels Enable Low-Maintenance Commercially Sized Rechargeable Zn–MnO_2_ Batteries for Use in Solar Microgrids

**DOI:** 10.3390/polym14030417

**Published:** 2022-01-20

**Authors:** Jungsang Cho, Gautam Ganapati Yadav, Meir Weiner, Jinchao Huang, Aditya Upreti, Xia Wei, Roman Yakobov, Brendan E. Hawkins, Michael Nyce, Timothy N. Lambert, David J. Arnot, Nelson S. Bell, Noah B. Schorr, Megan N. Booth, Damon E. Turney, Gabriel Cowles, Sanjoy Banerjee

**Affiliations:** 1 The Energy Institute, City College of New York, Steinman Hall, New York, NY 10031, USA; chojs0114@gmail.com (J.C.); lunaweixia@gmail.com (X.W.); brendan.e.hawkins@gmail.com (B.E.H.); mnyce1957@gmail.com (M.N.); megan@urbanelectricpower.com (M.N.B.); damonturney@gmail.com (D.E.T.); sanjoy@urbanelectricpower.com (S.B.); 2 Research & Development Department, Urban Electric Power, Pearl River, NY 10965, USA; meir@urbanelectricpower.com (M.W.); adupreti96@gmail.com (A.U.); chessrom1@gmail.com (R.Y.); gabe@urbanelectricpower.com (G.C.); 3 Sandia National Laboratories, Albuquerque, NM 87185, USA; tnlambe@sandia.gov (T.N.L.); djarnot@sandia.gov (D.J.A.); nsbell@sandia.gov (N.S.B.); nschorr@sandia.gov (N.B.S.)

**Keywords:** hydrogels, zinc, manganese dioxide, rechargeable, solar microgrid

## Abstract

Zinc (Zn)–manganese dioxide (MnO_2_) rechargeable batteries have attracted research interest because of high specific theoretical capacity as well as being environmentally friendly, intrinsically safe and low-cost. Liquid electrolytes, such as potassium hydroxide, are historically used in these batteries; however, many failure mechanisms of the Zn–MnO_2_ battery chemistry result from the use of liquid electrolytes, including the formation of electrochemically inert phases such as hetaerolite (ZnMn_2_O_4_) and the promotion of shape change of the Zn electrode. This manuscript reports on the fundamental and commercial results of gel electrolytes for use in rechargeable Zn–MnO_2_ batteries as an alternative to liquid electrolytes. The manuscript also reports on novel properties of the gelled electrolyte such as limiting the overdischarge of Zn anodes, which is a problem in liquid electrolyte, and finally its use in solar microgrid applications, which is a first in academic literature. Potentiostatic and galvanostatic tests with the optimized gel electrolyte showed higher capacity retention compared to the tests with the liquid electrolyte, suggesting that gel electrolyte helps reduce Mn^3+^ dissolution and zincate ion migration from the Zn anode, improving reversibility. Cycling tests for commercially sized prismatic cells showed the gel electrolyte had exceptional cycle life, showing 100% capacity retention for >700 cycles at 9.5 Ah and for >300 cycles at 19 Ah, while the 19 Ah prismatic cell with a liquid electrolyte showed discharge capacity degradation at 100th cycle. We also performed overdischarge protection tests, in which a commercialized prismatic cell with the gel electrolyte was discharged to 0 V and achieved stable discharge capacities, while the liquid electrolyte cell showed discharge capacity fade in the first few cycles. Finally, the gel electrolyte batteries were tested under IEC solar off-grid protocol. It was noted that the gelled Zn–MnO_2_ batteries outperformed the Pb–acid batteries. Additionally, a designed system nameplated at 2 kWh with a 12 V system with 72 prismatic cells was tested with the same protocol, and it has entered its third year of cycling. This suggests that Zn–MnO_2_ rechargeable batteries with the gel electrolyte will be an ideal candidate for solar microgrid systems and grid storage in general.

## 1. Introduction

Research into making alkaline zinc (Zn)–manganese dioxide (MnO_2_) batteries rechargeable has recently gained interest because these batteries are environmentally friendly, safe and the raw materials used are cheap and abundant. The active raw materials of MnO_2_ and Zn also have high theoretical specific capacities of 617 mAh/g and 820 mAh/g, respectively, which make an energy-dense battery [1,2,3,4]. These advantageous properties and characteristics have allowed the Zn–MnO_2_ battery to flourish as a primary battery for over 70 years. It is the most dominant primary battery chemistry because of its single discharge high energy density of >400 Wh/L which has been widely utilized in the household small electronics sector. However, if it can be made rechargeable, then its advantageous properties can be used for a wide range of applications that are in vogue such as long-duration storage, solar microgrids and uninterruptible power sources to serve the energy needs and climate action goals of the 21st century.

Rechargeable Zn–MnO_2_ alkaline batteries have been studied for the past 40 years with many excellent contributions [5]. These can be classified into two branches, where one branch has concentrated on developing novel strategies to improve performance [5,6,7] while the other has concentrated on uncovering fundamental insights on the electrochemical performance of Zn and MnO_2_ [8,9,10]. Many studies have been devoted to improving the electrode performance while few have concentrated on the electrolytes [11]. Recent work has studied changing the alkaline electrolyte to neutral or acidic electrolyte with reports of improved performance [12,13]. Although very interesting, the Zn–MnO_2_ battery performance with neutral or acidic electrolyte has only been shown at very low areal capacities and unfortunately has not translated to commercial use.

Rechargeable alkaline Zn–MnO_2_ batteries can be further classified into proton-insertion chemistry and conversion chemistry [14,15,16,17]. The Zn anodes exhibit conversion reactions where they dissolve and deposit during their 2e^−^ reactions delivering 820 mAh/g. The polymorph of MnO_2_ dictates if the battery is classified as proton-insertion or conversion. The electrolytic manganese dioxide (EMD, γ-MnO_2_) or gamma-MnO_2_ has a 2e^−^ theoretical capacity of 617 mAh/g which delivers its first electron capacity (308 mAh/g) through a proton-insertion reaction to form MnOOH and the its second electron (308 mAh/g) through a conversion reaction to finally form Mn(OH)_2_. On recharge, the Mn(OH)_2_ undergoes the 2e^−^ charge reactions to form another polymorph of MnO_2_ called birnessite or delta-MnO_2_ [18,19,20]. The delta-MnO_2_ was considered to be irreversible in the past because of the uncontrollable conversion reactions involving Mn^3+^ and Mn^2+^ ions, so much of the work was concentrated on making the 1e^−^ proton-insertion reaction rechargeable to 10% to 50% of its theoretical capacity (308 mAh/g) [21,22]. Recently, our group has demonstrated the complete 2e^−^ reversibility using additives such as bismuth oxide and copper, and further work is being conducted to commercialize this technology which involves the development of binders and improvement of Zn anode utilization [23,24]. The 1e^−^ technology also has commercial significance because of its higher nominal voltage (~1.3 V) compared to the 2e^−^ technology which is usually ~1 V. This manuscript will focus on the 1e^−^ proton-insertion chemistry of Zn–MnO_2_ with a future manuscript in preparation for the 2e^−^ technology.

The electrochemical reactions of the Zn–MnO_2_ battery chemistry are as follows:

Zn anode:

(1) $\mathrm{Zn}+4{\mathrm{OH}}^{-}\;↔\;\mathrm{Zn}{\left(\mathrm{OH}\right)}_{4}^{2-}+2e^{-}$ dissolution reaction
(2) $\mathrm{Zn}{\left(\mathrm{OH}\right)}_{4}^{2-}\;↔\;\mathrm{ZnO}+2{\mathrm{OH}}^{-}+H_{2}O$ deposition–precipitation reaction

MnO_2_ cathode:

(3) ${\mathrm{MnO}}_{2}+H_{2}O+e^{-}\;\to \mathrm{MnOOH}+{\mathrm{OH}}^{-}$ 1e^−^ reaction (proton insertion reaction)
(4) $\mathrm{MnOOH}+H_{2}O+e^{-}\;\to \mathrm{Mn}{\left(\mathrm{OH}\right)}_{2}+{\mathrm{OH}}^{-}$ 2e^−^ reaction (dissolution–precipitation reaction)

The failure mechanisms of the 1e^−^ Zn–MnO_2_ battery are due to the redistribution of zinc particles and passivation of the metallic Zn anode due to its dissolution and deposition–precipitation reactions. Mass transfer limitations and higher limiting current densities can lead to the formation of mossy and dendritic Zn particles that can short the battery [25,26]. MnO_2_ under higher utilizations (>50%) of its 1e^−^ capacity will undergo crystal structure breakdown and delamination from the current collector because of its high-volume expansion. Utilizations between 50 and 80% of the MnO_2_’s 1e^−^ capacity can lead to the formation of soluble Mn^3+^ ions that lead to the formation of inactive spinel Mn_3_O_4_. Soluble Zn ions reacting with the dissolved Mn^3+^ ions will also form inactive spinel of ZnMn_2_O_4_ or hetaerolite [27]. The liquid alkaline electrolyte can be considered as the true source of these failures. If the free movement of soluble zincate and Mn^3+^ ions can be curtailed, it could mitigate most of the failure modes in the 1e^−^ battery. Our proposed approach to solving these problems is the use of a gelled potassium hydroxide electrolyte.

Gelled electrolytes such as hydrogels are an alternative to liquid electrolytes and have been extensively used in batteries. Tuning the viscosity of gelled electrolytes can help to limit the crossover of soluble ions, reduce volume expansion, reduce occurrences of shorts and curtail Zn redistribution. In Zn-anode batteries, there have been some reports on using hydrogels. These reports have been vital in trying to improve the performance of Zn-anode batteries, but there have not been reports on specifically improving the 1e^−^ performance of Zn–MnO_2_ batteries. Moreover, very few reports have tried to understand the relationship between the utilization of Zn and MnO_2_’s theoretical capacity with the type of hydrogel (non-crosslinked and crosslinked) used in the battery.

In this manuscript, we comprehensively report on the fundamental mechanisms, performance improvements and commercialization aspects of using a hydrogel in a rechargeable Zn–MnO_2_ battery. We concentrate on using a potassium polyacrylate gel synthesized through the free radical polymerization route. From a fundamental standpoint, we report on the utilization of Zn and MnO_2_ capacity with the type of hydrogel used with further analysis from a rheological viewpoint. We demonstrate the benefits of using hydrogels to mitigate some of the common modes of failure seen in traditional liquid batteries such as preventing shape change, passivation and breakdown of the MnO_2_ electrode for many cycles. In terms of commercialization, we report on tailoring the gelation method and its parameters to perform in situ gelation so that this process can be scaled for commercialization. We report on the novel use of hydrogels to prevent overdischarge by chemically limit the utilization of the electrodes in a commercial cell and thus show a possibility of eliminating the use of battery management systems for this chemistry.

In terms of market applicability, we report on the performance of a full-scale battery system that was built in-house utilizing the hydrogels reported in this manuscript. The system is a 2 kWh system comprising 72 cells with one set (8 cells in parallel) and nine sets in series. This system was made to cycle using the IEC solar protocol 61,427, which is the standard for demonstrating a new chemistry or battery system’s viability for the solar microgrids. These systems are gaining importance as power outages start being a persistent issue in the United States because of its aging infrastructure being vulnerable to extreme climate events. With the demonstration of the gelled Zn–MnO_2_ system, we show it to be a viable alternative to toxic lead–acid battery systems and generators that are currently being deployed. The use of gelled electrolyte in this system also makes the Zn–MnO_2_ battery chemistry a low-maintenance and nonhazardous system for shipping based on the Department of Transportation’s classification standards.

## 2. Experiment

Electrolytic manganese dioxide (EMD) was purchased from Borman (Henderson, NV, USA). Zinc (Zn) powders were purchased from Grillo, Germany. Potassium hydroxide (KOH) was purchased from Fisher Scientific, Hampton, NH, USA. Graphite was purchased from TIMCAL, Bodio, Switzerland. Indium oxide, indium hydroxide, acrylic acid (AA), potassium persulfate (K_2_S_2_O_8_) and *N*,*N’*-methylenebisacrylamide (MBA) were purchased from Sigma-Aldrich, St. Louis, MO, USA. Nickel and copper meshes were purchased from Dexmet, Wallingford, CT, USA. Dispersion Teflon was purchased from DuPont, Wilmington, DE, USA.

### 2.1. Electrode Fabrication

Electrodes were made by first mixing the raw materials with dispersion Teflon into a homogeneous mixture and rolled into sheets using a rolling pin or a rolling machine. These sheets were dried at 60 °C overnight. The dried sheets were then pressed onto current collectors. Cathode sheets were pressed onto nickel mesh while anode sheets were pressed onto copper mesh. These were laboratory-made electrodes for laboratory-scale tests. Sometimes commercial-scaled electrodes were also used for laboratory-scale tests. The commercial-scaled electrodes were provided by Urban Electric Power (UEP) which used a proprietary coating process that was completely automated to coat electrode materials onto current collectors and calendar. The commercially scaled cells either in prismatic or cylindrical form factor were made using UEP’s electrodes.

The cathodes comprised 80 wt.% EMD, 16 wt.% graphite and 4 wt.% Teflon, while the anodes comprised 97 wt.% Zn and 3 wt.% Teflon. These compositions remained the same for the laboratory and commercially made cells.

The fabricated electrodes’ size changed with the type of experiment performed. Usually, the sizes ranged from 1.27 cm × 1.27 cm to 7.62 cm × 15.24 cm. Cyclic voltammetry tests were performed using 1.27 cm × 1.27 cm electrodes, while cycling experiments were performed using 2.54 cm × 2.54 cm, 5.08 cm × 7.62 cm and 7.62 cm × 15.24 cm sized electrodes.

### 2.2. Electrolyte Preparation

Two types of electrolytes were used for electrochemical tests. The controlled experiments used a liquid potassium hydroxide (KOH) solution, while the rest of the experiments used gelled KOH solution. The gelled KOH, which is a hydrogel, used liquid KOH as one of its main precursors, which will be described shortly. The liquid KOH was prepared by mixing KOH pellets with deionized water (DI water) until the desired concentration was obtained.

**Figure 1:**
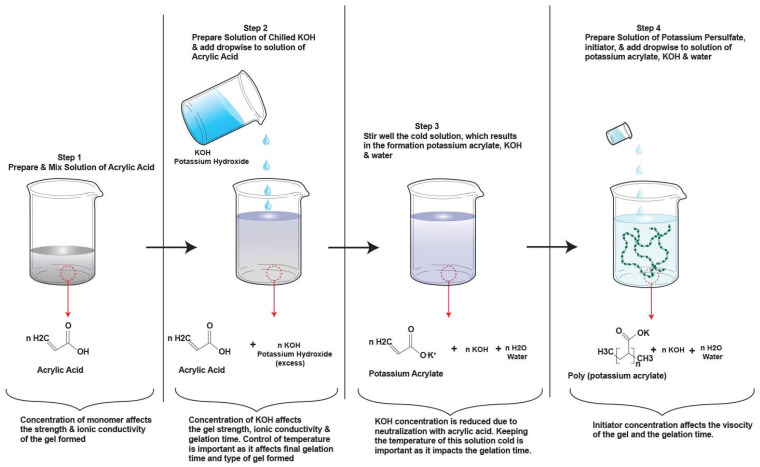
Schematic procedure showing the detailed steps for the free radical polymerization reaction of hydrogel electrolytes.

The hydrogel was synthesized through a free radical polymerization reaction as shown in Figure 1. 20 mL of Acrylic acid (AA) was added dropwise to the solution of 100 mL liquid KOH in a volumetric ratio of 1 to 5 to form a mixture of KOH and potassium acrylate (PA). This was done in an ice bath as adding AA to KOH will result in a temperature rise because it is an exothermic reaction. In some of our tests, we even added zinc oxide (ZnO) and indium oxide or hydroxide (In_2_O_3_ or In(OH)_3_) in this reaction to include gassing inhibiting additives. The effect of these additives will be explained later in the manuscript. The cooled neutralized mixture of KOH–PA was polymerized by adding 82 μL of potassium persulfate (KPS) initiator solution (4 wt.% with DI water). In some cases, we also crosslinked this hydrogel to test its effect on the electrochemistry of Zn–MnO_2_ batteries. The crosslinking was done by using 0.1 g *N*,*N’*-methylenebisacrylamide (MBA) which was dissolved in AA solution. This solution was then added dropwise to KOH or KOH with additives solution and the same procedure followed as aforementioned.

This was the general method used to synthesize our hydrogels. We further optimized this synthesis for large-scale commercialization of the Zn–MnO_2_ battery. For example, we tuned the synthesis to prolong the gelation time so that the electrolyte could be filled in a liquid state where the electrode materials could have enough time to soak. This will be further explained in the manuscript.

In the manuscript, we have also adopted “x” as a numerical factor for the KPS and MBA, where “x” for the KPS equates to 82 μL and for the MBA equates to 0.1 g. For example, if we have mentioned 2x for the initiator then we have used 164 μL of the 4 wt.% KPS solution.

### 2.3. Battery Fabrication

Prismatic and cylindrical form factor cells were tested. Laboratory tests were conducted in polysulfone boxes whose dimensions were 8.28 cm × 5.66 cm × 15.88 cm (width × depth × height). Electrode packs consisting of anodes and cathodes were wrapped in two layers of cellophane (0.00254 cm in each layer) separator. These packs were compressed in polysulfone boxes between polypropylene shims.

**Figure 2:**
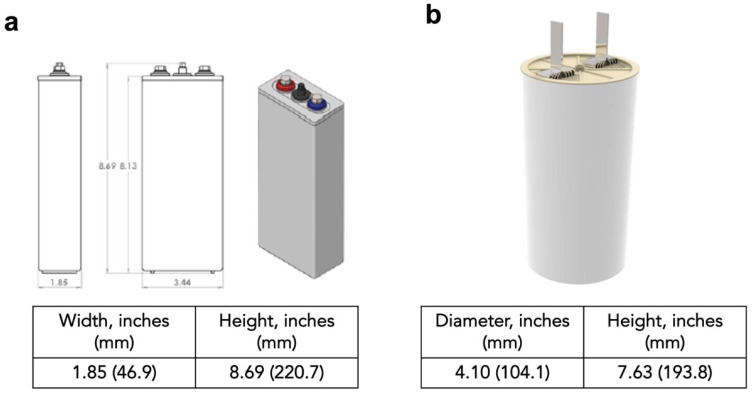
(**a**) Commercially made 19 Ah prismatic cell. (**b**) Commercially made 70 Ah cylindrical cell.

Commercially made cells were tested in prismatic and cylindrical form factors. The prismatic form factor cells contained 7.62 cm × 15.24 cm cathodes and anodes that were paired to form a 95 Ah designed capacity based on 1e^−^ capacity of MnO_2_. The cylindrical form factor cells contained long electrodes with cellophane separator that were rolled into jelly roll and fitted inside a container. A schematic of the cell designs and dimensions is shown in Figure 2.

### 2.4. Electrochemical Experiment and Characterization

A multichannel Arbin BT 2000 and PEC tester were used for galvanostatic experiments. Cyclic voltammetry and impedance experiments were performed on a Biologic potentiostat/galvanostat (VSP Modular 5-channel) instrument. For ex situ characterization, electrodes were washed and soaked in DI water and dried. XRD was done on a PANalytical X’Pert Pro Powder Diffraction instrument fitted with a PIXcel1D fast detector and Cu Ka filter.

### 2.5. Rheological Testing of Gel Properties

Studies of changes in rheological properties were performed using a stress-controlled Haake MARS rheometer system (Thermo Fisher), using a 60 mm 1° cone and a flat plate sensor. Temperature was controlled using a Phoenix recirculating bath system, and measurements at 10 and 25 °C were taken following sample equilibration. Kinetic studies of gelation via the KOH method used an oscillating time sweep program, with a 60 s preshear at 500 1/s, followed by a 15 s zero shear rate rest duration. The oscillation test was at a constant frequency of 1 Hz and a constant displacement of 0.002 rad. This was used to follow the development of gel properties over time durations varying from 2000 to 10,000 s. Gel formation was determined by the crossover point for G’ (elastic or storage modulus) and G’’ (viscoelastic or loss modulus), or alternatively the point where tan δ = 1 (where δ = G’’/G’).

### 2.6. Conductivity Measurements

Potentiostatic electrochemical impedance spectroscopy was used to measure the ionic conductivity of the gels. A disc 0.5 inches in diameter and approximately 2–3 mm thick was punched out from the gel and placed between two stainless steel rod electrodes in a Swagelok cell. Impedance was measured on the cell using a Biologic MPG-205 potentiostat from 20 kHz to 100 mHz with a sinusoidal amplitude of 10 mV from open-circuit potential. The bulk resistance was taken as the intercept of the Nyquist plot with the real impedance axis, found by extrapolating a linear fit of the 10 highest frequency points. Conductivity was calculated using the formula

$$\sigma =\frac{\tau }{R_{b}A}$$

where σ is the ionic conductivity, τ is the thickness, $R_{b}$ is the bulk resistance and A is the cross-sectional area.

### 2.7. Gassing Measurement and Bubble Activity Measurement

Ten grams of Zn was added to a glass tube, followed by 5 mL of electrolyte. The Zn powder and electrolyte were briefly mixed with a stir bar, and then the tube was filled with mineral oil. A j-tube was inserted into the cylinder, and the system was then placed in a water bath at 45 °C for 1 h. The oil level was then zeroed and recorded. The tube rested in the water bath for 3 days, after which the final oil level was recorded. The gas rate (μL H_2_/g Zn/day) was then calculated from these values.

Zn electrodes with the dimensions 1 in × 1 in were placed on a petri dish and the pre-gelation KOH liquid was poured onto the electrodes to completely cover it. These anodes were completely encapsulated once the KOH liquid had gelled. After 2 h, the petri dish was placed under a microscope and the rate of bubbles being formed was visually analyzed for 5 min. The number of bubbles passing through the surface of the gel was counted. After two days, the petri dishes were placed under the microscope again and the bubbles were counted again.

## 3. Results and Discussion

### 3.1. In Situ Polymerization Process and Hydrogel Development

The hydrogels or polymer electrolytes reported in the literature are free-standing films that are placed on electrodes of low areal capacity and cycled. The polymerization process can be described as ex situ as it is a different step compared to the cell assembly. Commercialization of these cells is impractical and unfeasible. Another drawback is the low interfacial contact area between the porous electrodes and the gelled electrolyte, which leads to poor active material utilization and rate capability. Our motivation and primary aim were to develop a synthesis procedure that develops hydrogels that retain the properties of the ex situ films, such as good mechanical strength (to develop leakproof cells), good chemical and electrochemical stability for long cycles and ionic conductivity, and can be made into a continuous process for large-scale manufacturing. This entails the creation of an in situ polymerization process where the solution is first filled in the cell in its liquid state and then gelled over time. This allows increased and better contact area between the gelled electrolyte and porous electrodes. The synthesis procedure for the hydrogels is described in detail in the experimental section and schematically depicted in Figure 1, where we have used a free-radical polymerization process.

**Figure 3:**
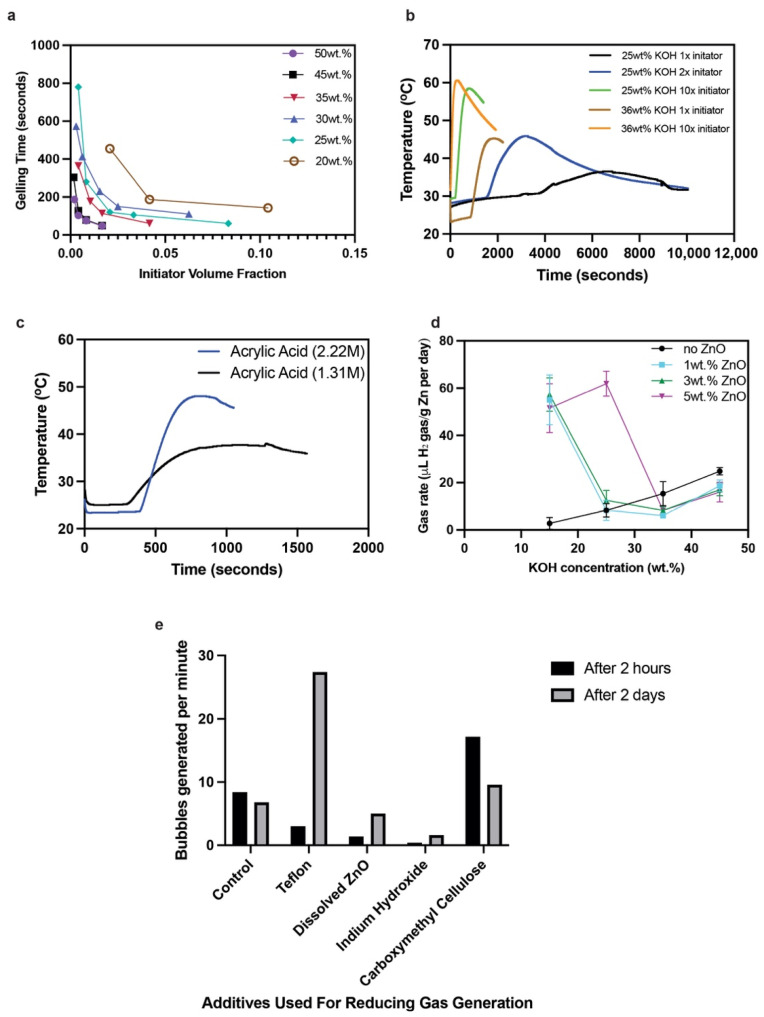
(**a**) The effect of KOH concentration (see legend) and initiator concentration on polymerization rate. In the experiments, 4 wt.% of initiator was used. (**b**) Effect of initiator concentration on the polymerization rate. (**c**) Effect of monomer (acrylic acid) concentration on the polymerization rate. (**d**) Effect of dissolved ZnO amount on the gassing rate of the Zn powder. (**e**) Effect of electrolyte additives in the hydrogel electrolyte on the hydrogen generation, which is measured through bubble activity.

The schematic process in Figure 1 describes the important parameters that determine the polymerization rate of the free radical polymerization process. Temperature, monomer (acrylic acid), initiator (potassium persulfate) and electrolyte (KOH) concentration influence the polymerization process. First, we studied the impact of initiator volume fraction (initiator solution/total solution) and KOH concentration on gelation time, as shown in Figure 3a. The results showed that reducing the initiator and KOH concentration significantly lowered the polymerization rate and resulted in longer gelation times, which is required for successful in situ polymerization. The lowered polymerization rates could be due to two factors: reduction of potassium acrylates after the neutralization step and reduction of persulfate radicals (S_2_O_8_^2−^) which are needed for initiating the polymerization reactions. Potassium acrylates are the monomers after the initial neutralization step that polymerize to create a hydrogel with KOH. Reductions of KOH and initiator concentrations result in a slower monomer conversion rate process, which is essential for in situ polymerization. Another factor in the lowering of the polymerization rate is the temperature, where in the experiments, the solutions were chilled to 0 °C. The solutions were chilled because the neutralization reaction of acrylic acid and KOH is exothermic. This exothermic or self-heating predetermined the maximum monomer concentration that could be used in the experiment (acrylic acid could not exceed 25 wt.% of the total solution) as it could lead to violent and rapid self-accelerating polymerization reactions. Lowering the temperature also influenced the final physical structure of the gel, where gels synthesized at lower temperatures had a higher viscosity while the gels polymerized at room temperature had lower viscosity. Higher viscosities are needed for creating a leak-proof battery cell.

The effects of initiator and monomer concentrations on the polymerization rate were further studied by measuring the time to temperature rise and peak temperature obtained in the polymerization process. In these experiments, the potassium acrylate–KOH solutions synthesized after Step 3 were allowed to equilibrate at room temperature to study these parameter effects. For the effect of initiator concentration, polymerization rates were increased when the initiator amount was increased 10 times from its original value, as shown in Figure 3b. The polymerization was instant for 25 and 36 wt.% KOH concentration, which showed that there were enough S_2_O_8_^2−^ radicals to propagate the reaction. However, at the original initiator amount (1x), the effect of KOH concentration had an impact on the time to initiate the polymerization and the polymerization rate. For 1x initiator amount, the time to initiate the polymerization process for 25 wt.% KOH was >4 times than that of the 36 wt.% KOH. The time to initiate the polymerization process can also be termed as “soaking time” for the solution before the polymerization process. The longer the soaking time or time to initiate polymerization, the better the infiltration of the prepolymer solution will be into the pores of the electrodes. The reduction in the polymerization rate, i.e., reduction in the temperature increase, is also good for in situ polymerization as the gelation would proceed over hours to form a viscous gel with well-developed branches of the polyacrylate chains that contain water and KOH. A higher portion of free water content in these hydrogels is important for hydroxyl diffusivity, which is important for high ionic conductivity and electrochemical mechanisms of both the MnO_2_ and Zn electrodes. The reduction in polymerization rate was also seen when the monomer concentration was reduced from the controlled value of 2.22 M of acrylic acid to 1.31 M (initiator was kept at 1x and 36% KOH). These experiments showed that for obtaining longer soaking times during the in situ polymerization process and its eventual gelation, it is important to reduce the amount of initiator, reduce the monomer concentration and vary the KOH concentration to obtain the desired hydrogel. Rheology is also important when developing the right hydrogel, which will be discussed later in the manuscript.

Zn anodes corrode in alkaline electrolytes to release hydrogen (H_2_) gas. Moreover, during the charging step, dissolved Zn ions (called zincate, Zn(OH)_4_^2−^) electrochemically plate to form metallic Zn (−1.35 V vs. Hg–HgO). However, hydrogen evolution reactions also take place as side reactions that reduce the Coulombic efficiency and increase the loss of active materials from the electrode. These would also happen in polymer hydrogel electrolytes unless additives are included in the solution before polymerization. We first studied the gassing rate of Zn powder in controlled liquid electrolytes of varying concentrations from 15 to 45 wt.%. The gassing rate increased linearly in the controlled electrolytes. To reduce the gassing or corrosion, studies in the past have reported the use of ZnO dissolved in the electrolyte. We tried the same strategy of dissolving various ZnO concentrations from 1 to 5 wt.%, which is its saturation amount, and measured the H_2_ gassing rates, as shown in Figure 3d. The trend was not clear in this case as lower KOH concentrations showed a considerable increase in gassing compared to the control. The reasons are still unclear but it could be attributed to low ZnO solubility in low KOH concentrations which would mean its effect is very minimal; however, it looks like it acts as a catalyst for hydrogen evolution. In higher KOH concentrations such as 36 wt.%, it is very clear that saturating the electrolyte with ZnO reduces the H_2_ gassing compared to the control. As 5 wt.% ZnO is the saturation amount in 36 wt.% KOH, it prevents any further self-discharge or corrosion of metallic Zn powder.

We further measured the hydrogen generation of the Zn anodes embedded in the hydrogel electrolyte. We conducted these experiments under a microscope as we were unable to use our traditional gas measuring apparatus because of the gelled (viscous) nature of the electrolyte. These experiments were conducted under controlled temperature and pressure. Our initial observation with the controlled electrolytes containing no additives was that H_2_ bubbles would form on the surface of the Zn anode in the gelled electrolyte and rise through the viscous electrolyte. Therefore, we measured H_2_ bubbles per minute as a metric as this was easier to do with the naked eye and monitor it. We measured the bubble activity after 2 h of the first gelation step with Zn anodes embedded in it and after 2 days. The various additives tried in this experiment are shown in Figure 3e. Teflon and carboxymethyl cellulose were tried because of their fibrous nature and Teflon’s hydrophobic nature that would allow H_2_ gas to pass through the gel easily. However, these additives acted as a catalyst for H_2_ generation, and the bubble generation rate was still unusually high after 2 days. We tried ZnO and indium hydroxide as other additives and found that these two additives individually succeeded in reducing the corrosion rate of the anodes even in gelled electrolyte. The reduction was considerable even for the controlled sample, as shown in Figure 3e; therefore, we decided on including saturated ZnO and indium hydroxide as combined additives in the solution during the gelation process.

**Figure 4:**
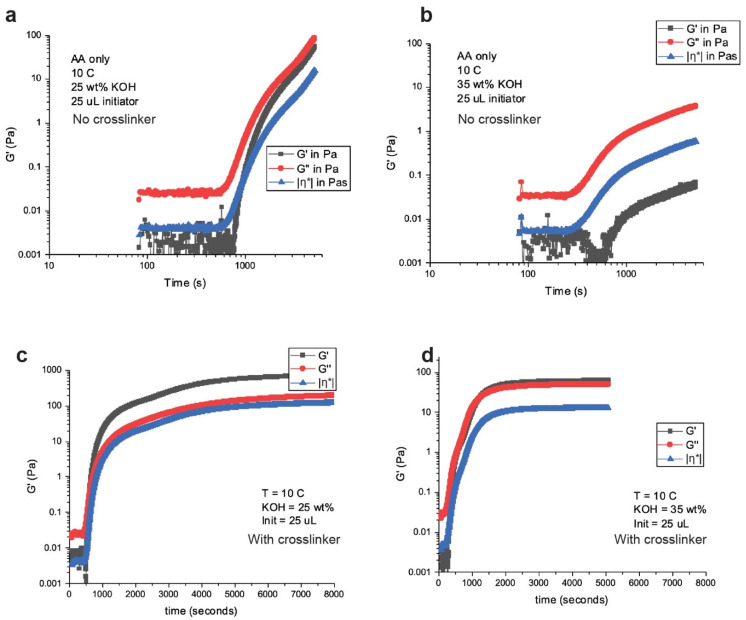
Measurement of storage and loss modules over time of (**a**) non-crosslinked 25 wt.% KOH, (**b**) non-crosslinked 35 wt.% KOH, (**c**) crosslinked 25 wt.% KOH and (**d**) crosslinked 35 wt.% KOH.

The physical robustness of these gels is important to create a leak-proof cell. Leak-proof cells or batteries are desired for transportation purposes as it reduces safety concerns. Department of Transportation classifies a “nonhazardous” cell or battery as one that is nonspillable or leak-proof, which means that on cell cracking there should not be any flow of electrolyte. Liquid electrolyte cells fail this test. Gelled electrolytes can pass these tests as they are “robust” in nature or viscous enough that they do not flow. Crosslinkers are used in polymerization synthesis to increase the strength of polymers. MBA is a crosslinker used in free radical polymerization synthesis [28,29]. We also tried this crosslinker, and in our initial observations, we found consistent results with gels without crosslinker when testing for delaying the initiation time to polymerization and reducing the polymerization rate. The crosslinker does not affect the polymerization kinetics. We measured the rheological properties of crosslinked and non-crosslinked gels at 25 and 35 wt.% KOH concentrations. These results are shown in Figure 4.

The gelation map in Figure 4 shows the elastic (G’) and viscoelastic (G’’) modulus behavior of non-crosslinked and crosslinked gels. The –η*– is the complex viscosity of the gels. The transition from liquid to solid is signified by the crossover of the G’ and G’’, where the polymers are truly described as “gels”. The non-crosslinked gels in Figure 4a,b cannot be described as true “gels”; however, they are very viscous fluids still showing hydrogel behavior. The crosslinked gels in Figure 4c,d have G’ and G’’ crossover signifying they are “gels”. Another behavior noticed from the non-crosslinked and crosslinked gels are the “strength” of the gels, where the higher KOH concentrations are “weaker” gels compared to the lower KOH concentrations. The viscosity of the crosslinked 25 wt.% KOH gel was 722 Pa while the 35 wt.% KOH gel was 64 Pa. Stronger or more viscous gels may initially signify a positive attribute; however, in our gas generation studies, we found that stronger gels, especially crosslinked gels, trapped H_2_ bubbles which lead to swelling of the hydrogels and build-up of pressure.

**Figure 5:**
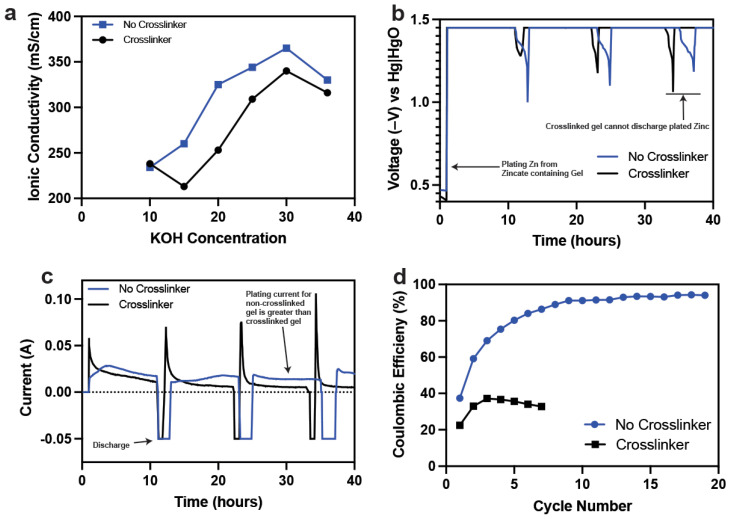
(**a**) Ionic conductivity comparison between non-crosslinked and crosslinked gels. (**b**) Galvanostatic charge and discharge performance of Zn anodes in non-crosslinked and crosslinked gels. (**c**) Current behavior on charge and discharge of electroplated Zn anodes in non-crosslinked and crosslinked gels. (**d**) Coulombic efficiency comparison of anodes in non-crosslinked and crosslinked gels.

Electrochemical characteristics of non-crosslinked and crosslinked gels were also studied. The electrochemical work was based on efficient Zn plating. The MnO_2_ in the 1e^−^ range is proton intercalation, while the Zn discharges and charges its 2e^−^ capacity through dissolution–precipitation reactions. Dissolution–precipitation reactions depend on free water content and the efficient movement of zincate ions. If zincate ion movement is impeded or dissolution of Zn ions is impeded, then the active material utilization from the Zn anode would be curtailed, which would impact battery performance. We studied the gel ionic conductivity and Zn plating characteristics in non-crosslinked and crosslinked gels, as shown in Figure 5.

The ionic conductivity of the non-crosslinked and crosslinked gels was in the same range, as shown in Figure 5a. These conductivities matched that of liquid electrolyte. The ionic conductivity of these gels is the highest reported in the literature compared to other electrolyte systems such as acidic and neutral-based. This meant that hydroxyl transport in these gels was not impeded and the electrochemical performance of a Zn–MnO_2_ was possible. The crosslinked gels had a minor drop in conductivity which could be because of rigid domains formed within the hydrogel by crosslinked bonds.

The electrochemical performance of a Zn anode was tested in these hydrogels because of Zn’s dissolution–precipitation behavior. An electrochemical cell was constructed where the hydrogels contained ZnO dissolved in it. A copper current collector was used as the working electrode and sintered nickel as the counter electrode. The working electrode was controlled and monitored using a mercury–mercury oxide (Hg–HgO) reference electrode. The aim of the experiment was to test the plating behavior of Zn from the hydrogels (non-crosslinked and crosslinked) onto the metallic current collector. The electrolyte contained 2 Ah of capacity and the experiment was based on cycled 10% of it. The first step in the experiment was to apply a constant voltage at −1.4 V from OCV to plate Zn from the hydrogels (see Figure 5b). On achieving the charge capacity, the anode was discharged until −1 V to measure the Coulombic efficiency.

The charge and discharge characteristics shown in Figure 5b–d indicate the considerable reduction in Zn ion mobility in the crosslinked hydrogels. Zn movement is severely hampered, which affects the plating of metallic Zn. This is indicated by the current decay behavior in Figure 5c. The current reaches its most minimum value for the crosslinked gels while the non-crosslinked gels sustain a constant higher plating current. The Coulombic efficiency of the crosslinked gels never crosses 40%, indicating limited Zn-ion diffusion and side reactions dominating the coulombs on charge. The crosslinked hydrogels developed trapped H_2_ bubbles which eventually swelled.

Considering the reaction rate study, gassing behavior, rheological behavior and electrochemical plating and discharge performance of Zn anodes, we concluded a non-crosslinked hydrogel formed with low initiator and monomer concentration with 36 wt.% KOH as the final concentration was the best for our in situ polymerization process and further battery performance study. The high KOH concentration also helps to dissolve more ZnO and indium hydroxide (In(OH)_3_) as corrosion inhibitors. From this point in the manuscript, all the hydrogels are non-crosslinked containing 37 ± 1 wt.% KOH as the final concentration and ZnO and In(OH)_3_ dissolved in it.

### 3.2. Electrochemical Performance of Zn–MnO_2_ in In Situ Polymerized Gels

The electroanalytical and battery performance of the Zn–MnO_2_ cells containing the optimized hydrogel electrolyte was tested and compared to similar cells containing liquid electrolyte (control cells). Cell construction and method of filling are written in the experimental section.

**Figure 6:**
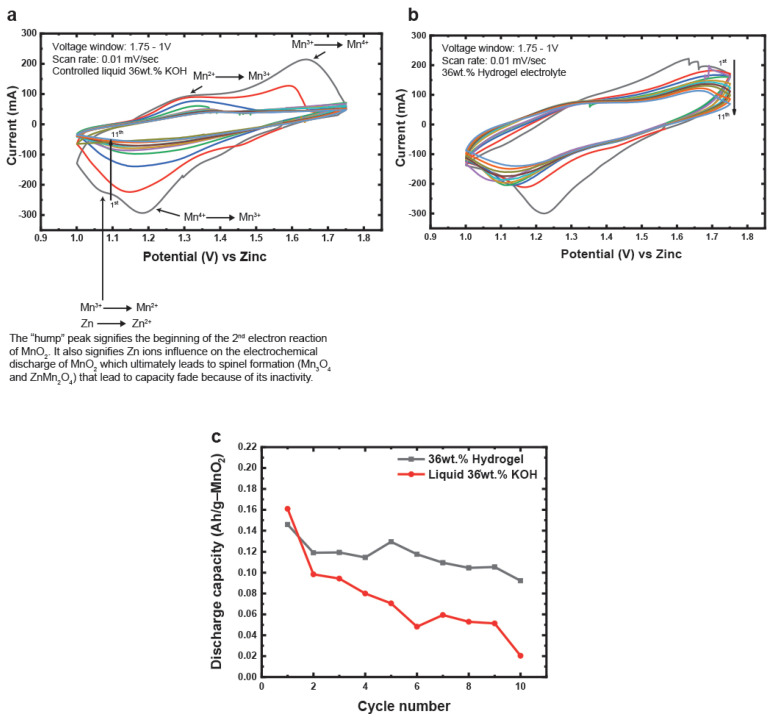
(**a**) Cyclic voltammetry curve of a Zn–MnO_2_ cell containing liquid 36 wt.% KOH scanned at 0.01 mV/s between 1.75 V and 1 V. (**b**) Cyclic voltammetry curve of a Zn–MnO_2_ cell containing hydrogel 36 wt.% KOH scanned at 0.01 mV/s between 1.75 V and 1 V. (**c**) Capacity retention comparison of Zn–MnO_2_ cells containing either liquid or hydrogel 36 wt.% KOH.

Cyclic voltammetry tests were first conducted to understand the redox reactions of Zn and MnO_2_ electrodes in hydrogel electrolyte and its comparison to liquid electrolytes. The cells were scanned at 0.01 mV/s between 1.75 V and 1 V to obtain detailed peaks during the reduction and oxidation reactions. In the liquid electrolytes, well-defined reduction and oxidation peaks are seen in the first cycle, as shown in Figure 6a. Each of these well-defined peaks is characterized according to its respective reactions. Since there is no capacity limit in cyclic voltammetry tests, the MnO_2_ electrode reacts at a greater extent in its first electron region. Mn experiences dissolution of its Mn^3+^ ions in the reduction step when >50% of its 1e^−^ capacity is accessed [30]. Beyond 79% of its 1e^−^ capacity, a large portion of the Mn^3+^ ions undergo side reactions to form inactive spinel structures such as hausmannite (Mn_3_O_4_) [31]. Zn ion mobility is also very high in 36 wt.% KOH solutions. Zn forms dissolved zincate ions (Zn(OH)_4_^2−^) during discharge which precipitate to form ZnO. However, these dissolved Zn^2+^ ions also migrate to the cathode side to poison or undergo side reactions with Mn^3+^ ions to form another inactive spinel called hetaerolite (ZnMn_2_O_4_). These reactions are marked in Figure 6a, where the “hump” or the second reduction peak in the first is characterized. The ease of accessibility or high utilization of the electrode active materials in high liquid KOH concentrations leads to buildup of spinel structures in subsequent cycles and leads to capacity loss, which is seen by the reduction in peak area. Comparing the same scanning rates for an identical cell made with in situ polymerized hydrogel electrolyte (Figure 6b), there is no peak that signifies the reduction of Mn^3+^ to Mn^2+^ or the interaction of Zn^2+^ with Mn^3+^. The peak area for the hydrogel-containing cell also appears to reduce slightly in subsequent cycles compared to its liquid counterpart. This signifies better retention in capacity. Capacity retention of these cells was calculated, as shown in Figure 6c, where the superior retention of the hydrogel-containing cell is a clear advantage to the control liquid cell. The better capacity retention in hydrogel electrolyte can be attributed to a few reasons such as reduced Mn^3+^ dissolution and reduced loss or migration of dissolved zincate ions from the Zn anode. These points will be discussed later in the manuscript.

**Figure 7:**
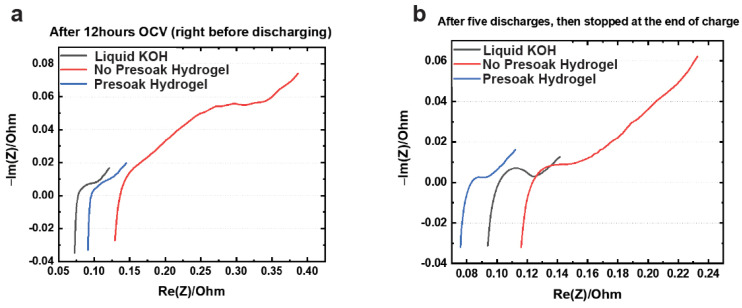
(**a**) EIS spectra at open-circuit voltage (OCV) of Zn–MnO_2_ cells containing liquid KOH and hydrogel. The hydrogel-containing cells had two variations. The cells were presoaked with liquid KOH electrolyte and decanted before gelling or were directly gelled with no presoaking. (**b**) EIS spectra of Zn–MnO_2_ cells containing liquid and hydrogel cycled 5 times. The hydrogel-containing cells had two variations. The cells were presoaked and decanted before gelling or cells were directly gelled with no presoaking.

Electrochemical impedance spectroscopy (EIS) was also done to understand better the evolution of solution and charge transfer resistance with cycling. These results are shown in Figure 7. At open-circuit voltage (OCV), the EIS spectra of a cell containing liquid KOH are better compared to those of a cell containing hydrogel in terms of its solution resistance and charge transfer resistance (semicircle), as shown in Figure 7a. This could be due to longer soaking times available for a cell containing liquid electrolyte where there is much better interfacial contact with the electroactive materials and KOH. This is also seen after five discharge cycles where the solution resistance is higher for the hydrogel-containing cell. There is no difference in capacity retention between these cells, but it is an indication that in much later cycles these resistances can build up to affect the capacity retention, especially at much higher utilization of the electrode active materials. We solved this problem by making another cell which was presoaked with liquid KOH for 12 h and decanted before the gelling procedure. After gelation of a presoaked cell, the EIS spectra are very similar to those of a cell containing liquid electrolyte at OCV and after five discharge cycles, as shown in Figure 7a,b. After noticing this effect, we presoaked our cells with liquid electrolyte before gelation. A way of eliminating this presoaking step is by increasing the initiation time to gelation to >10 h, which was possible with potassium acrylate as the monomer starting point.

**Figure 8:**
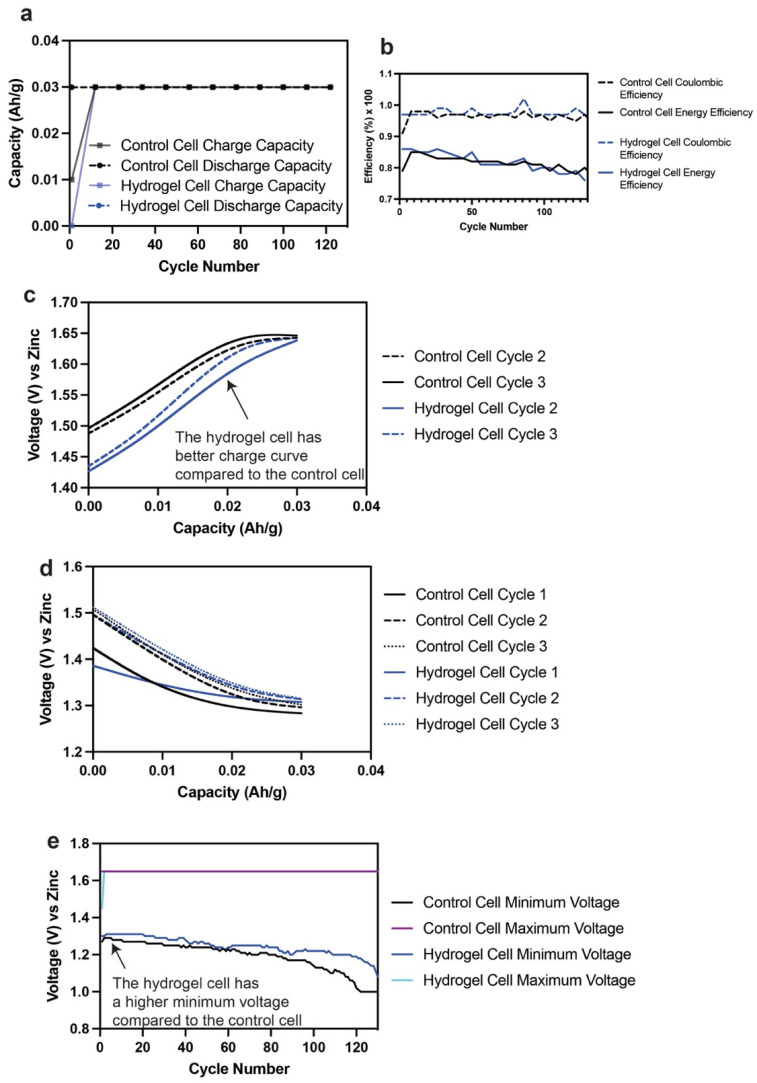
(**a**) Capacity retention comparison of a control (liquid) and hydrogel-containing Zn–MnO_2_ cell at 10% of MnO_2_ 1e^−^ capacity. (**b**) Coulombic and energy efficiency comparison of a control (liquid) and hydrogel-containing Zn–MnO_2_ cell at 10% of MnO_2_ 1e^−^ capacity. (**c**) Charge curves for cycles 2 and 3 of a control (liquid) and hydrogel-containing Zn–MnO_2_ cell at 10% of MnO_2_ 1e^−^ capacity. (**d**) Discharge curves for cycles 1, 2 and 3 of a control (liquid) and hydrogel-containing Zn–MnO_2_ cell at 10% of MnO_2_ 1e^−^ capacity. (**e**) Maximum and minimum voltage comparison of a control (liquid) and hydrogel-containing Zn–MnO_2_ cell at 10% of MnO_2_ 1e^−^ capacity.

The cycling performance of Zn–MnO_2_ cells containing liquid or hydrogel electrolyte was also compared, as shown in Figure 8. The cells were cycled at 10% utilization or depth of discharge (DOD) of the MnO_2_ 1e^−^ capacity (308 mAh/g) at C/2 (C = 30.8 mAh/g). The voltage ranges used were 1.65 V as the end of charge and 1 V as the end of discharge. The capacity retentions for the cells in liquid and hydrogel electrolyte were the same, as shown in Figure 8a. This was good as it showed that the hydrogel electrolyte did not impact cycling performance. The Coulombic efficiency of these cells was near 100%, while the energy efficiency was >80% until 130 cycles, as shown in Figure 8b. This showed that the hydrogels did not affect the nominal or average charge and discharge voltage during cycling. The charge and discharge curves for the first three cycles are shown in Figure 8c,d. The hydrogel-containing cell has a lower charge voltage than the liquid-containing cell, indicating a lower resistance to charge the cell. This advantageous characteristic is clearly seen in Figure 8e when comparing the minimum voltage fade for both the cells. The hydrogel-containing cell has a higher minimum voltage compared to its liquid counterpart, indicating its efficient charge transfer during cycling.

**Figure 9:**
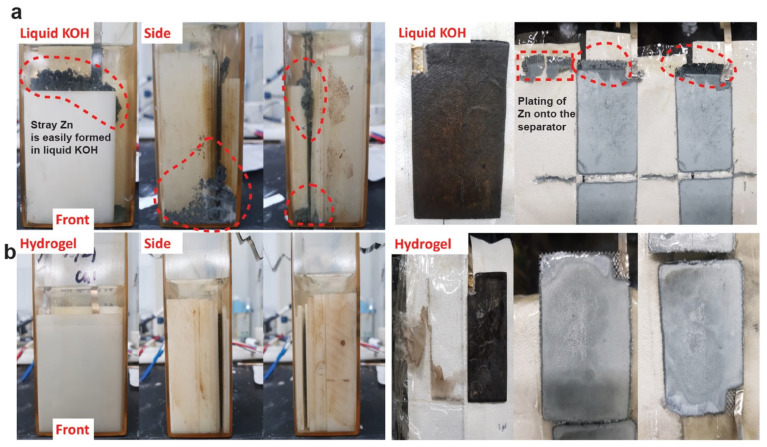
(**a**) Photographs of a Zn anode exhibiting considerable shape change during cycling in a Zn–MnO_2_ cell containing liquid 36 wt.% KOH. On the right side, pictures of dissected Zn and MnO2 electrodes are shown. Zn plating is also seen on the cellulose separator. (**b**) Photographs of a Zn–MnO_2_ cell containing 36 wt.% hydrogel electrolyte. There is no stray Zn or shape change seen. The electrodes on the right side are also free of any dendritic growth.

The hydrogel-containing Zn–MnO_2_ cell clearly showed advantages during cycling. These advantages were also noticeable on visual inspection of the cell. Zn anodes undergo a phenomenon called “shape change” where they dissolve and replate or precipitate in different regions of the electrode, leading to considerable active material redistribution. This can lead to the formation of stray Zn particles that are not connected to the metallic current collector. This was easily seen in the cells containing liquid 36 wt.% KOH, as shown in Figure 9a. The creation of these stray Zn particles is a loss of active material and thus a loss of capacity which is seen in our cycling cells. These stray Zn particles are also seen to form dendritic structures on the edges of the electrodes and on the separator, as shown in Figure 9a. These can also cause Coulombic inefficiencies by directing some of the coulombs on charge to side reactions and thus inefficient plating of Zn. These problems were not observed in the cells containing hydrogel electrolyte, as shown in Figure 9b. There was no stray Zn formation or formation of any dendritic Zn structures. The hydrogel electrolyte seemed to control shape change, a longstanding problem in metal anode batteries.

**Figure 10:**
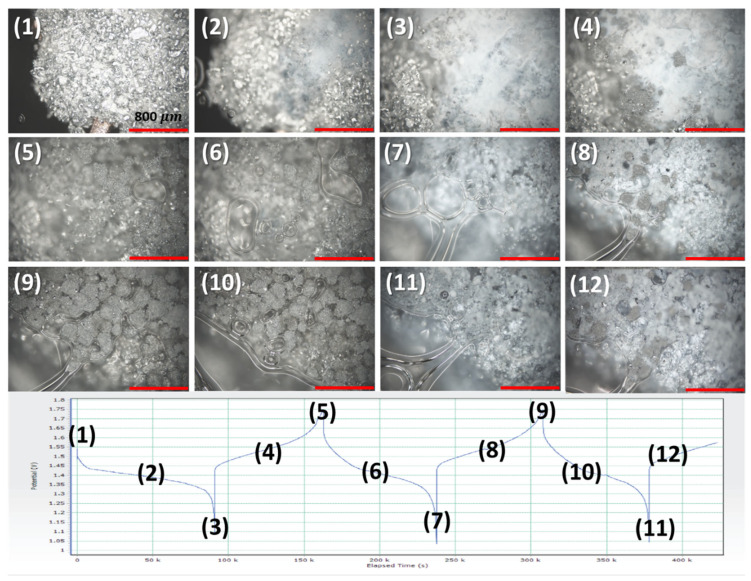
In operando optical microscope images of a Zn anode cycled in a hydrogel-containing Zn–MnO_2_ cell. Charge–discharge curves of the cell are shown in the bottom part with numbers denoting the regions where the pictures were captured. For video, please refer to Supplementary Video S1. All images have the same scale (scale bar = 800 μm).

To understand the hydrogel’s effect on deterring Zn shape change, we constructed small cells that could be cycled under an optical microscope. Cell construction and electrode architecture details are written in the experimental section. A Zn anode was cycled and observed in operando under the microscope, as shown in Figure 10. The cell contained hydrogel electrolyte encapsulating the Zn anode. The cell was cycled to obtain the maximum capacity of the Zn anode in hydrogel electrolyte while its electrode and particle changes were observed under the microscope. The numbers on the images represent the regions on the voltage–time curves where the images were taken. The video of this cell is also shown in Supplementary Video S1. During the first discharge (regions 1 to 3), the metallic Zn powder was seen to transition to ZnO (white powder appearance in region 3). The total Zn utilization in this discharge was >60% of its 2e^−^ capacity (820 mAh/g). This is remarkable utilization for a metallic anode in hydrogel electrolyte because the hydrogel reduces the diffusion of zincate ions, which could be expected to promote precipitation of ZnO and passivation of the electrode. Instead, despite ZnO precipitation, we observe high utilization and excellent reversibility, discussed below. The Zn utilization or overdischarge of Zn anodes could be curtailed because of limited electrolyte in the cell or reduced free water content in the gels, an advantage that we exploited and describe later in the manuscript.

On charge, from region 4 to region 5, the ZnO particles were reduced to form metallic Zn. The metallic Zn particles look to form domains within the hydrogel network which are tightly compressed. The hydrogel’s advantageous characteristic of blocking shape change was seen from regions 5 to 8, where at the end of discharge (regions 7 and 8), the ZnO particles looked to be encapsulated within the hydrogel network as well. The transition from region 8 to region 9 showed that the ZnO particles converted reversibly back to metallic Zn in the same regions. The hydrogel encapsulating the Zn particles is shown in region 10. The hydrogels can be viewed as a local reaction space where it preserves the charge and discharge end-products of conversion metal anodes such as Zn. At the end of discharge 3, the Zn utilization was >50% of its 2e^−^ capacity (820 mAh/g), which is very high.

### 3.3. Testing of Hydrogel-Containing Cells That Are Commercially Sized

**Figure 11:**
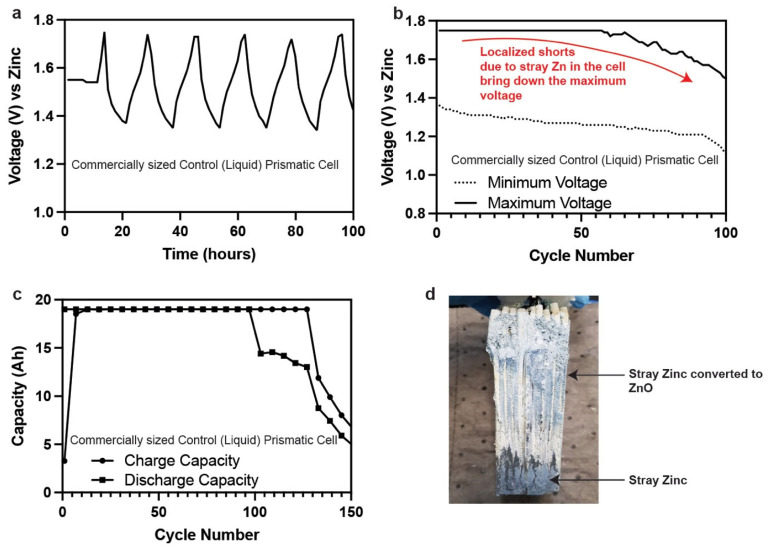
(**a**) Cycling curves for a commercially made Zn–MnO_2_ cell containing 36 wt.% liquid KOH nameplated at 19 Ah. (**b**) Maximum and minimum voltage fade for a commercially made Zn–MnO_2_ cell containing 36 wt.% liquid KOH. (**c**) Capacity retention of the control commercially made Zn–MnO_2_ cell. (**d**) Photograph of the dissected control commercially made Zn–MnO_2_ cell.

The microscope experiments showed the shape change limiting characteristic of the hydrogel electrolytes, and the promising cycling results motivated the next series of experiments, i.e., testing the hydrogels in commercially sized prismatic and cylindrical cells. First, we created a baseline performance of the prismatic cells in liquid 36 wt.% KOH. The total 1e^−^ capacity of the commercially made prismatic cells equated to 95 Ah. The cells were nameplated at 20% utilization (19 Ah). The cells were cycled at C/4 (C = 19 Ah) between 1.75 V and 1 V. The battery performance of this control cell is shown in Figure 11. The initial cycles showed that the cells charged and discharged coulombically at 100% (Figure 11a,c). However, in later cycles, the end of charge voltage slopes downwards with the end of discharge voltage, as shown in Figure 11b. This trend is a clear indication of a short being developed in the cell. Localized shorts in the cell direct the coulombs on charge to side reactions rather than to the active materials, which creates a cumulative coulomb deficit. At some point, this deficit impacts the discharge capacity.

This impact on the discharge capacity was seen on cycle 100 where there is rapid capacity degradation. Dissection of this cell (Figure 11d) showed that there was a considerable build-up of stray Zn that created these localized shorts which eventually led to the failure of the control (liquid) cell. In a liquid cell, this can be mitigated, and cycle life can further be increased by reducing the electrolyte concentration to 25 wt.% and using calcium hydroxide membranes [32].

**Figure 12:**
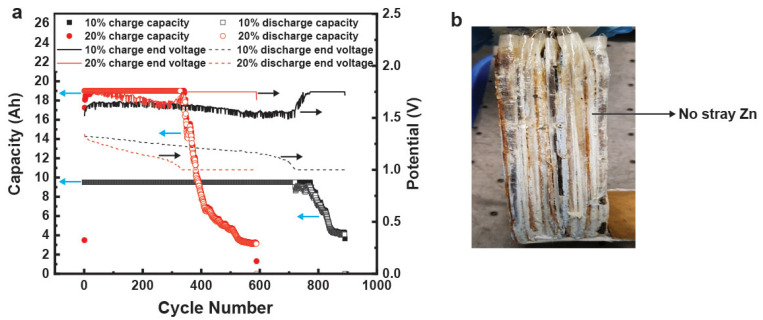
(**a**) Capacity retention and maximum and minimum voltage fade for a commercially made Zn–MnO_2_ cell containing 36 wt.% hydrogel electrolyte nameplated at 19 Ah. (**b**) Photograph of a dissected commercially made Zn–MnO_2_ cell containing 36 wt.% hydrogel electrolyte.

The analogous hydrogel-containing commercial prismatic cells were cycled to the same protocol as the control liquid cell. One cell was cycled at 10% utilization (9.5 Ah) and the other was cycled at 20% utilization (19 Ah). These hydrogel-containing cells obtained 100% DOD for numerous cycles, as shown in Figure 12a. The 9.5 Ah cell had 100% retention of capacity for >700 cycles while the 19 Ah cell had 100% capacity retention for >300 cycles, as shown in Figure 12a. The 19 Ah hydrogel-containing cell had a 3 times better cycle life compared to its liquid counterpart. This is the first such demonstration of a low-maintenance nonspillable Zn–MnO_2_ rechargeable aqueous battery. The dissection of these cells (Figure 12b) indicated that a possible reason for the considerable increase in cycle life was the reduction of stray Zn particles that lead to shorts and reduction in active material utilization.

**Figure 13:**
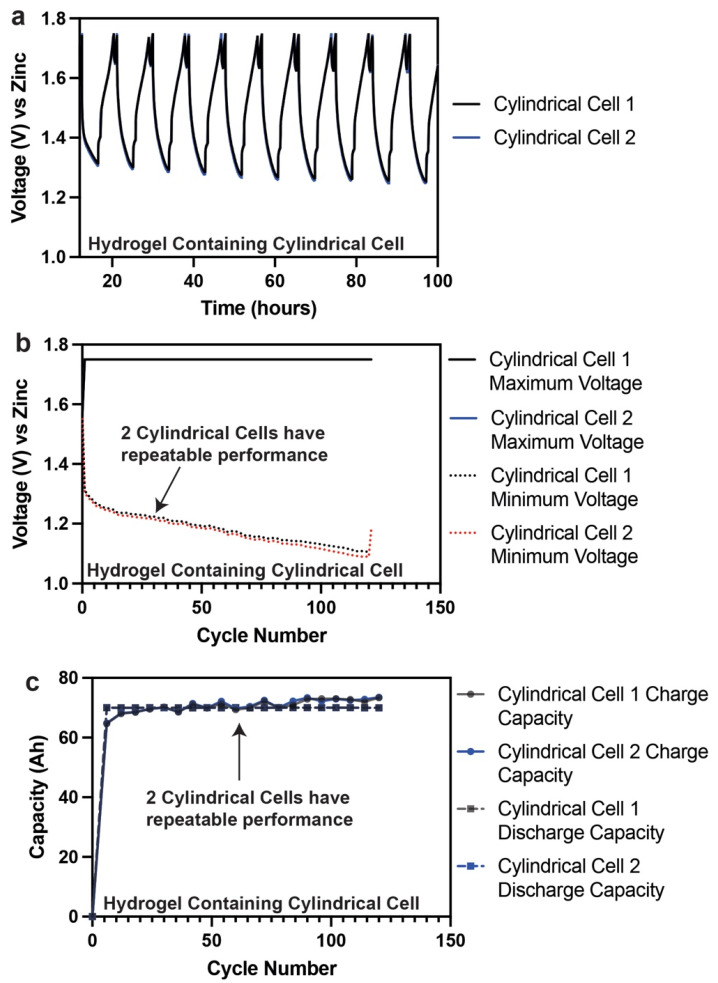
(**a**) Cycling curves for a commercially made cylindrical Zn–MnO_2_ cell containing 36 wt.% hydrogel nameplated at 70 Ah. (**b**) Maximum and minimum voltage fade for a commercially made cylindrical Zn–MnO_2_ cell containing 36 wt.% hydrogel nameplated at 70 Ah. (**c**) Capacity retention for a commercially made cylindrical Zn–MnO_2_ cell containing 36 wt.% hydrogel nameplated at 70 Ah.

Commercially made cylindrical cells containing hydrogel electrolyte were also tested. The cylindrical cells are nameplated at 70 Ah which equates to 20% utilization of MnO_2_ 1e^−^ capacity. Two cylindrical cells were tested with hydrogels formed using the in situ polymerization process. These cells were also cycled at C/4 (C = 70 Ah) between 1.75 V and 1 V. The results are shown in Figure 13. The two cylindrical cells had remarkable consistency in their performance. The voltage curves overlapped, as shown in Figure 13a. The maximum and minimum voltage fades were also consistent with each other, as shown in Figure 13b. The capacity retention of these cells was also 100%, as shown in Figure 13c. These cells are still cycling at the time of this writing and have crossed >120 cycles. The use of cylindrical cells allows for better compression and increasing the capacity of the cell or battery module, which eventually relates to system performance. The use of hydrogel electrolytes showed that these cells can be cycled reliably.

### 3.4. Overdischarge Protection and Commercial Applications of Hydrogel-Containing Zn–MnO_2_ Cells and Batteries

A major motivation for the current work was the development of nonspillable low-maintenance Zn–MnO_2_ cells that can be deployed in the field. Safety during system operation is important; however, safety during transportation is also important, and these hydrogel electrolytes help us satisfy the DOT requirements. From an application standpoint, we will discuss the use of these hydrogel-filled cells in a solar microgrid operation. However, there is also an important discussion point related to the operation of the cells in solar microgrids, uninterruptible power sources (UPSs), frequency regulation and other applications where overutilization of the cells can lead to side reactions that affect system energy and lifetime costs. A battery management system (BMS) is usually required to prevent the overcharge or the overdischarge of the cells and allow battery balancing. BMS can be expensive depending on the cell chemistry and add substantially to the system’s cost. If safety can be inbuilt from the chemistry side of the battery, it can lead to considerable benefits such as reducing system design complexity and costs.

**Figure 14:**
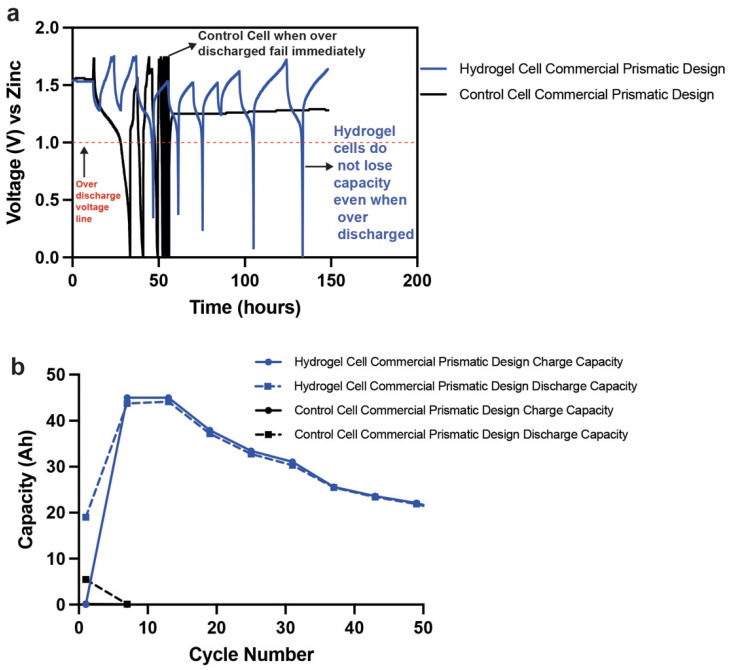
(**a**) Comparison of a control (liquid) and hydrogel-containing Zn–MnO_2_ prismatic cell that is overdischarged. (**b**) Capacity retention of a control (liquid) and hydrogel-containing Zn–MnO_2_ prismatic cell that is overdischarged.

We tested our hydrogels’ capacity to prevent overdischarge of Zn–MnO_2_ cells. Traditional Zn–MnO_2_ cells operated in the 1e^−^ range have a strict end of discharge voltage of 1 V. Crossing this 1 V discharge limit in a liquid-containing cell can lead to deleterious side reactions that affect cell rechargeability. As previously discussed, Mn^3+^ and Zn^2+^ ions can react together to form spinel compounds that are inactive and lead to rapid capacity deterioration. An example of this rapid capacity fade is shown in Figure 14a. Control commercial cells of Zn–MnO_2_ were overdischarged beyond 1 V. The total capacity achieved in this overdischarge was 130 Ah, which is considerable compared to the nameplate capacity (19 Ah) and theoretical 1e^−^ capacity (95 Ah). This meant that the cell accessed the second electron capacity, which is based on dissolution–precipitation of Mn ions. The uncontrollable nature of Mn ions in the second electron range and low voltage capacity is a main reason for curtailing the end of discharge of the 1e^−^ cell to 1 V. The Mn ions form [Mn–OH] octahedral complexes that react with each other and Zn ions to form spinels that cannot be recharged. This loss of rechargeability leads to immediate failure of the cell, as shown in Figure 14a,b.

We tested the same protocol on a hydrogel-containing commercial Zn–MnO_2_ cell and compared the overdischarge performance (Figure 14a). The hydrogel cell had no second electron reactions of MnO_2_ below 1 V and Zn ion interaction with the Mn below 1 V. The hydrogel was suppressing the dissolution of Mn and Zn ions that could lead to the formation of the inactive spinels. We postulate that this could be related to the free water content available in the hydrogels that allows for these reactions. The Zn anode utilizes a large portion of its theoretical capacity in liquid KOH, which leads to the dissolution of the active material to form zincate ions and then leads to the buildup of stray Zn in successive charge–discharge cycles. However, the same anode in hydrogel can be prevented from overdischarge by limiting the solubility of Zn in the hydrogels. The blocking of the Zn anodes’ overdischarge in turn prevents further discharge of even the cathodes and thus eliminates any deleterious side reactions. This can be done multiple times, as shown in Figure 14a,b, where continuously overdischarging to 0 V has no serious consequence on the capacity fade of the cell. Approximately 47% of the 95 Ah is obtained stably for 10 cycles, after which it experiences minor fade. This fade is due to the high utilization of the MnO_2_ and Zn anode beyond the stated 20% of 1e^−^ of MnO_2_.

Thus, we have shown an important benefit of these hydrogels, where we can tune the properties to impart safety protections chemically which has not been shown before.

From an application standpoint, we simulated the use of the hydrogel-containing cells in solar or photovoltaic off-grid applications. The solar off-grid application protocol is defined by the International Electrotechnical Commission (IEC) 61427-1, where it tests the batteries for use with photovoltaic energy systems that are used as constant, variable or intermittent energy to power equipment such as housing appliances (refrigerators, lighting) and pumps. For many communities or islands there is no access to the grid, so developing these systems with rechargeable batteries is a way to grant accessibility to power.

**Figure 15:**
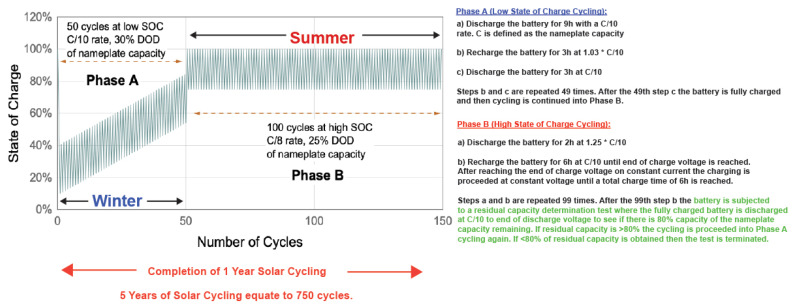
Schematic explanation of the IEC solar off-grid protocol. The protocol is done at 40 °C.

The IEC solar off-grid protocol is a demanding test protocol that subjects the cells or batteries to different states of charge at 40 °C and daily cycling. The different states of charge relate to the seasonal conditions or periods of time where there is no solar irradiation, and the battery system is required to supply energy for several days. For example, low solar irradiation is experienced during winters while high solar irradiation is experienced during summers. When there is low solar irradiation, the battery is operated at low state-of-charge (SOC) and when there is high solar irradiation the battery is operated at high state-of-charge. The IEC solar off-grid protocol specifically tests the cycling endurance of battery systems in photovoltaic applications and charge retention of the battery system after finishing one cycling loop (winter and summer cycles). A plot explaining the protocol is shown in Figure 15.

As explained in Figure 15, Phase A represents the winter cycling loop or low SOC, while Phase B represents the summer cycling loop or high SOC. The first step in Phase A is to discharge the battery 90% of its nameplate capacity at C/10 (where C is the nameplate capacity) to bring the battery to its low SOC. The battery is cycled in this low SOC 49 times in Phase A with a 3% overcharge in every recharge cycle. After finishing Phase A, the battery is recharged completely before proceeding into Phase B cycling. Phase B cycling is repeated 100 times until a total of 150 cycles is reached which concludes 1 year of solar cycling. Before Phase A is repeated, a residual capacity determination test is conducted to measure the percentage capacity retention of the battery with respect to its nameplated capacity. This is done at C/10 until the end of discharge voltage is reached. If >80% of the nameplate capacity is achieved, then the next year of solar cycling is started with the battery restarting at Phase A. If <80% of the nameplate capacity is achieved, then the testing is terminated. The aim for any battery manufacturer is to obtain maximum solar cycles. For example, 5 years of solar cycling would equate to 750 cycles or 10 years of solar cycling would equate to 1500 cycles at low and high SOC. Commercially available lead–acid (Pb–acid) batteries achieve at least 5 years in this protocol depending on the type of Pb–acid batteries used. Valve-regulated Pb–acid batteries can achieve >5 years but are very expensive, while flooded Pb–acid batteries are known to fail earlier.

**Figure 16:**
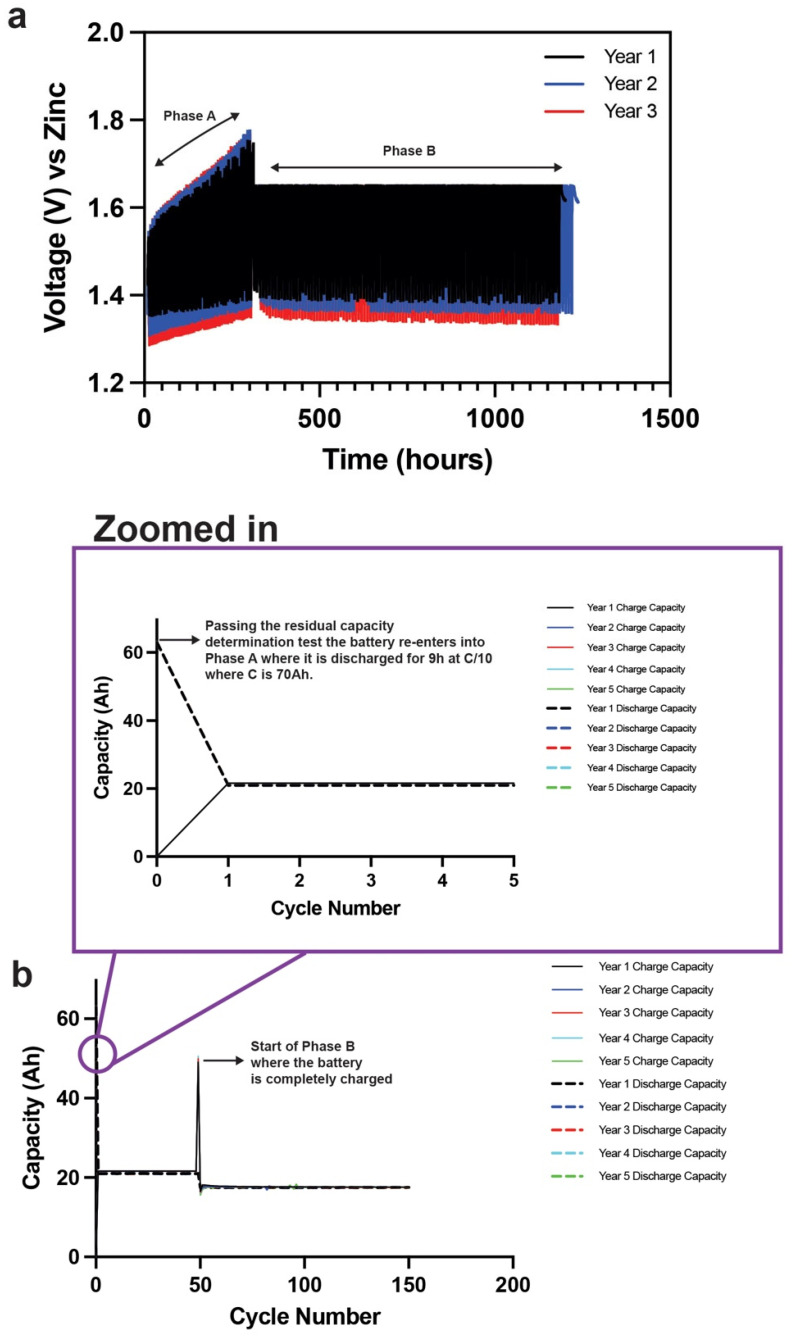
(**a**) Voltage–time curves of a commercial cylindrical 70 Ah Zn–MnO_2_ cell containing hydrogel electrolyte cycled using the IEC solar off-grid protocol. (**b**) Capacity retention of the hydrogel-containing 70 Ah cylindrical cell passing through Phase A and B 5 times, thus equating to 750 total cycles. The cell is still cycling in its 6th year.

For our Zn–MnO_2_ cells, in Phase A the end of charge and discharge voltages were 1.75 V and 1 V. This meant that the voltage would “walk” up the SOC curve during the 3 h recharged cycles until it hit 1.75 V by the 50th cycle. The end of charge voltage was then reduced to 1.65 V for Phase B to see if the required capacity could be injected into the battery at a lower voltage. The end of discharge voltage in Phase B was kept at 1 V. The nameplate was decided by the form factor used. For example, the cylindrical cells are nameplated at 70 Ah, while the prismatic cells are nameplated at 19 Ah. We cycled an individual cylindrical cell nameplated at 70 Ah filled with in situ polymerized hydrogel electrolyte under the IEC solar off-grid protocol, as shown in Figure 16. The hydrogel cylindrical cell has passed 5 years of solar cycling which equates to 750 cycles through Phase A and Phase B. The cell has entered its sixth year and still cycling well. The voltage–time curves are shown in Figure 16a, where the curves are remarkably consistent through numerous cycles of low and high SOC. The cell passed the residual capacity determination test after every year. Every year’s initial 9 h C/10 discharge at the start of Phase A is shown in the zoomed-in image in Figure 16b. The cell discharged 63 Ah of the 70 Ah nameplate capacity to enter its low SOC and walk up the SOC curve at the end of Phase A. The cell was completely charged before entering Phase B, as shown in Figure 16b. The cell passed through 750 cycles of different SOCs, which showed the robustness of the hydrogel electrolyte in the cylindrical cells.

**Figure 17:**
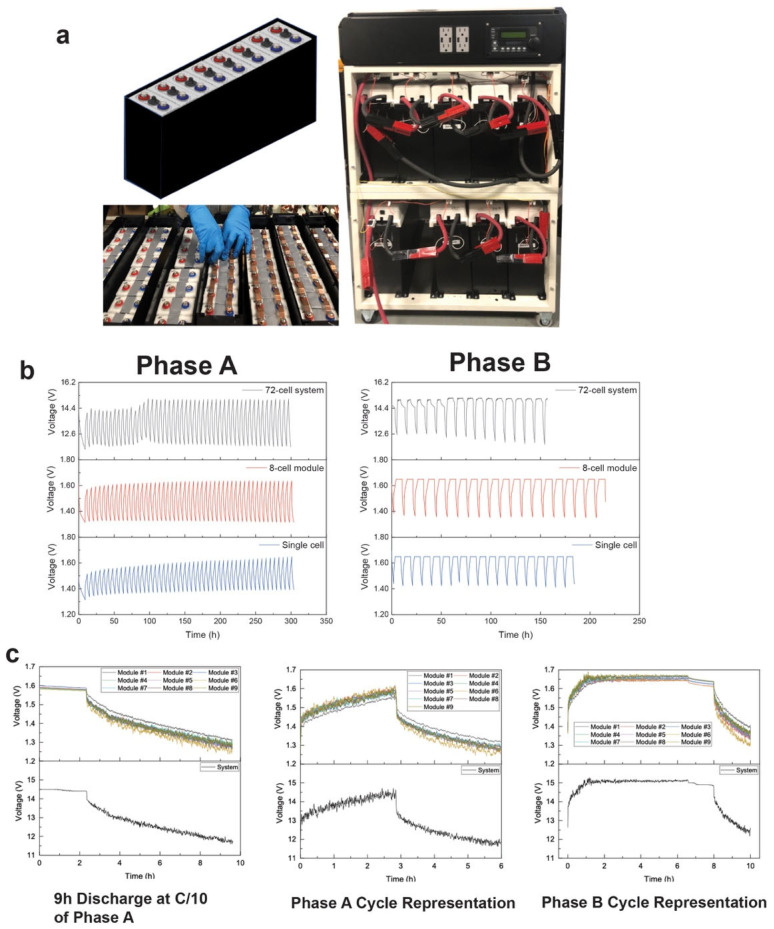
(**a**) A 2 kWh cube system constructed at UEP containing 72 prismatic cells filled with hydrogel electrolyte. (**b**) Comparison of a single prismatic cell, a module containing 8 cells connected in parallel and the 2 kWh system in Phase A and Phase B cycling regimes. (**c**) Comparison of the voltage curves of each module and the overall system at different stages—the first 9 h discharge at C/10 in Phase A, a representative Phase A cycle and a representative Phase B cycle.

The promising results of the commercially designed prismatic and cylindrical cells motivated our next experiment on system design and testing the hydrogel-containing cells in series and parallel. This is part of UEP strategy for a power assurance system for home power backup, where we are designing cube systems that can be combined into multiple cube containers to tailor the system energy and power according to the customer’s needs. We designed a 12 V system with 72 prismatic cells (nameplated at 19 Ah), where nine modules were connected in series and each module contained eight prismatic cells connected in parallel. Photographs of the module and system build are shown in Figure 17a. The system was again tested according to the IEC solar off-grid protocol through Phase A and Phase B cycling regimes. The system was nameplated at 2 kWh based on ~19 Ah per cell. To track the replicability of the system curves, we made a single cell and an 8-cell module connected in parallel and compared its cycling curves to that of the system, as shown in Figure 17b. The cycling curves were remarkably replicable for a single cell, eight-cell module in parallel and a 2 kWh system in both Phase A and Phase B cycling regimes.

We also monitored each module’s voltage during the system cycling using an Orion BMS. The individual module voltages and the overall system voltage are shown in Figure 17c. From right to left, the module cell voltages matched those of the system when comparing the first step in Phase A, i.e., the 9 h C/10 discharge; a representative cycle of Phase A; and a representative cycle of Phase B. The hydrogel-containing prismatic cells showed very consistent performance even in the constant voltage steps of Phase B. In control liquid cells, we have found that the constant voltage step results in earlier failure of Zn–MnO_2_ cells because of stray Zn formation that results in shorts and other side reactions that are an effect of the reducing float current. These problems were not seen in the cells containing hydrogel electrolyte. The system has gone through 2 years of cycling and has entered its third year.

## 4. Conclusions

An in situ polymerization method to develop hydrogels is reported that enhances contact with the electrode active material and reduces corrosion and loss of active material. These hydrogels reduce zincate migration, reduce formation of stray Zn particles and reduce manganese dissolution to increase the utilization of the electrode materials in the 1e^−^ cycling region of Zn–MnO_2_. The hydrogels also enhance the safety by reducing dendrite formation and stray Zn formation that often lead to short circuits or loss of Zn active material in the cell. These hydrogel-containing cells are also “nonspillable” and low-maintenance (no additional electrolyte is required during the battery’s lifetime), meeting the DOT requirements for safe transportability of a “nonhazardous” battery. These hydrogels show great promise for 1e^−^ Zn–MnO_2_ batteries; however, future work needs to be conducted on applying these gels for 2e^−^ Zn–MnO_2_ batteries, where both electrodes undergo conversion reactions. The 2e^−^ batteries are highly energy-dense because both electrodes are cycled at their maximum utilization. It will be interesting to study how these hydrogels can maintain their maximum utilizations when the solubility of the active material ions is reduced. This can lead to promising directions in tailoring the polymer gel properties, which is exciting for the field.

The development of the in situ polymerization method allowed for continuous manufacturing of commercially sized prismatic and cylindrical cells. We found through testing of these cells that hydrogels not only increased cycle life but also imparted other features such as overdischarge protection by reducing the solubility of zincate ions. This chemical method of imparting built-in safety in the cell using hydrogels is the first demonstration reported in battery literature. We identified an application space for the gelled Zn–MnO_2_ cells in the solar off-grid market. Toxic and unsafe Pb–acid batteries dominate this space. We found that our hydrogel-containing Zn–MnO_2_ cells can outperform the Pb–acid batteries by cycling through 5 years of the harsh IEC solar off-grid protocol. We further tested these hydrogels in a 2 kWh power assurance system designed by UEP which showed remarkable consistency in performance to a single cell and has passed through 2 years of solar cycling and has entered its third year. A low-maintenance nonspillable safe Zn–MnO_2_ battery system could provide myriad benefits to the public at large by increasing power accessibility in remote locations and mitigating the worst impacts of power outages in our increasingly connected society.

## Data Availability

The data presented in this study are available on request from the corresponding author. The data are not publicly available due to it being Urban Electric Power’s data.

## Supplementary Materials

The following supporting information can be downloaded at https://www.mdpi.com/article/10.3390/polym14030417/s1, Video S1: In operando optical video of a Zn anode cycled in hydrogel-containing Zn-MnO_2_ cell.
